# Therapeutic effects of the PKR inhibitor C16 suppressing tumor proliferation and angiogenesis in hepatocellular carcinoma *in vitro* and *in vivo*

**DOI:** 10.1038/s41598-020-61579-x

**Published:** 2020-03-20

**Authors:** Takao Watanabe, Hiroko Ninomiya, Takashi Saitou, Sota Takanezawa, Shin Yamamoto, Yusuke Imai, Osamu Yoshida, Ryosuke Kawakami, Masashi Hirooka, Masanori Abe, Takeshi Imamura, Yoichi Hiasa

**Affiliations:** 1 Department of Gastroenterology and Metabology, Graduate School of Medicine, Ehime University, Toon, Ehime Japan; 2 Department of Molecular Medicine for Pathogenesis, Graduate School of Medicine, Ehime University, Toon, Ehime Japan; 3 Translational Research Center, Ehime University Hospital, Toon, Ehime 791-0295 Japan; 4 Department of Lifestyle-related Medicine and Endocrinology, Graduate School of Medicine, Ehime University, Toon, Ehime Japan

**Keywords:** Hepatocellular carcinoma, Molecular medicine

## Abstract

The therapeutic effects of C16, which is an inhibitor of RNA-dependent protein kinase (PKR), on growth of hepatocellular carcinoma (HCC) cells and tumor progression *in vitro* and *in vivo* were evaluated. Huh7 cells, a human HCC cell line, were used. The effects of C16 on cell viability were evaluated with the MTT assay, and real-time RT-PCR was performed. Huh7 cells were grafted into immunodeficient mice, and the *in vivo* effects of C16 on tumorigenesis were examined. C16 suppressed proliferation of HCC cells in a dose-dependent manner *in vitro*. Mouse models with xenograft transplantation showed that the inhibitor suppressed the growth of HCC cells *in vivo*. Moreover, C16 decreased angiogenesis in HCC tissue in the xenograft model. Consistent with these results in mice, transcript levels of vascular endothelial growth factor-A and factor-B, platelet-derived growth factor-A and factor-B, fibroblast growth factor-2, epidermal growth factor, and hepatocyte growth factor, which are angiogenesis-related growth factors, were significantly decreased by C16 *in vitro*. In conclusion, the PKR inhibitor C16 blocked tumor cell growth and angiogenesis via a decrease in mRNA levels of several growth factors. C16 may be useful in the treatment of HCC.

## Introduction

Hepatocellular carcinoma (HCC) is one of the more common causes of cancer-related mortality, with approximately 748,300 new cases and 695,900 deaths globally each year^1,2^. Risk factors for HCC include hepatitis virus infection, exposure to aflatoxin B, alcohol consumption, and metabolic disorders such as obesity, diabetes, and hemochromatosis^3,4^. Hepatitis B virus (HBV) and hepatitis C virus (HCV) infections affect a large proportion of individuals worldwide. Approximately 150 million people are currently infected globally, with around 3–4 million new cases of infection annually^5^. Infection with HCV is a major cause of HCC^6^.

Double-stranded RNA-dependent protein kinase (PKR) is a serine/threonine protein kinase that is expressed throughout the body. PKR was first reported to be an anti-viral protein in the innate immune system that is induced by interferon^7^. HCV replication stimulates numerous proteins including PKR. PKR plays an important role in the anti-viral and anti-proliferative effects of interferon^8^. PKR modulates multiple signal transduction pathways, including the mitogen-activated protein kinase (MAPK), signal transducer and activator of transcription (STAT)^9,10^, and nuclear factor kappa-light-chain-enhancer of activated B cells (NF-κB) pathways^11^. PKR also transduces signals that lead to apoptosis^12,13^. Thus, PKR plays multiple functional roles in the regulation of inflammatory and immune signaling^14^.

To clarify the role of PKR in HCV-related hepatocarcinogenesis, we previously examined the phosphorylation of PKR in paired malignant and surrounding non-malignant tissues from patients with HCV-related HCC and showed that PKR protein levels are consistently increased in HCV-related HCC tissue compared with surrounding non-HCC tissue^15^. We modulated PKR expression in the HCV-replicating cell lines JFH-1 and H77 (generated by Huh7.5.1 cell transfection) using siRNA and a PKR expression plasmid, and we showed that PKR upregulates c-Fos and c-Jun activities through activation of extracellular signal-regulated kinase 1/2 (ERK1/2) and c-Jun N-terminal protein kinase (JNK1), respectively, subsequently increasing HCC cell proliferation^16^. We also confirmed the coordinated expression of c-Fos and c-Jun with PKR in human HCC specimens with HCV infection. The amounts of c-Fos and c-Jun and their phosphorylation levels are increased in specimens with high levels of PKR expression.

Although our previous reports showed that modulating endogenous PKR can affect HCC progression *in vitro*, therapeutic strategies targeting PKR in HCC have not been examined. The aim of this study was to investigate the therapeutic effects of the PKR inhibitor oxindole/imidazole compound (C16) on HCC cell growth and tumor progression *in vitro* and in an *in vivo* xenograft transplantation model to determine whether PKR inhibitors have potential as new therapeutic agents for HCC.

## Results

### PKR inhibitor C16 suppressed proliferation of HCC cells in a dose-dependent manner *in vitro*

**Figure 1:**
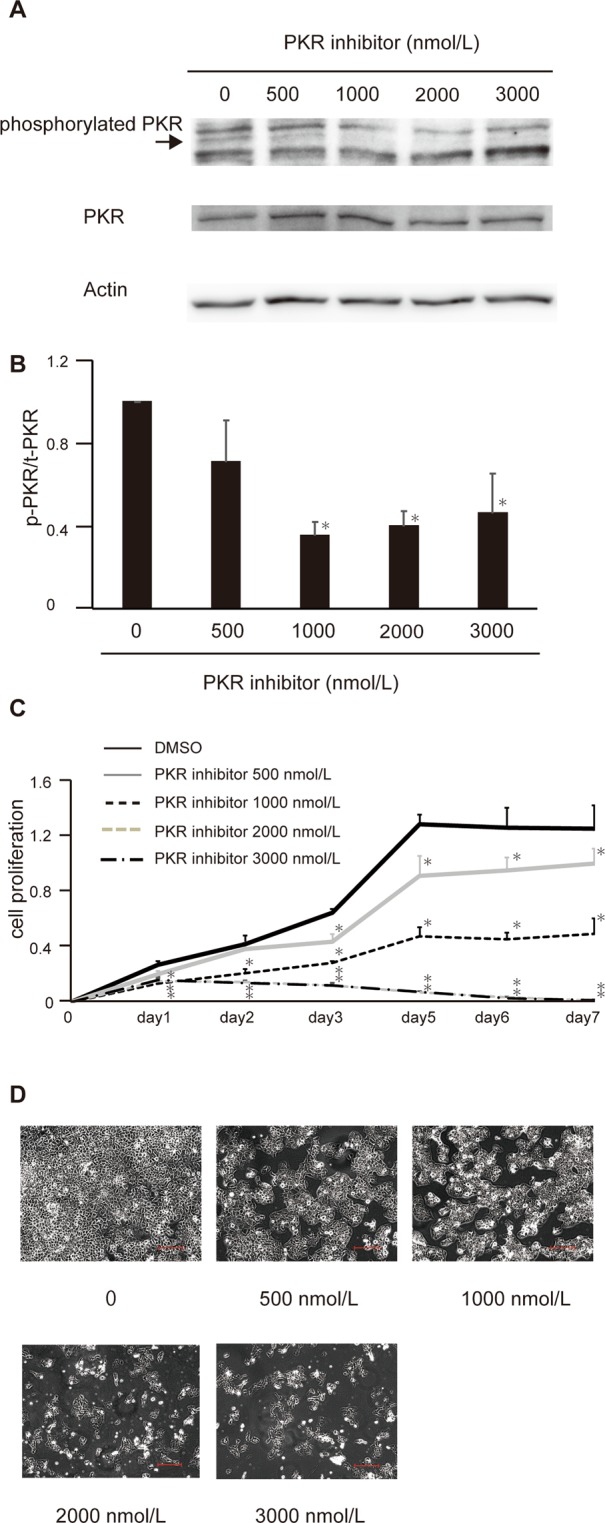
PKR inhibitor treatment suppresses HCC cell proliferation in a dose-dependent manner *in vitro*. Huh7 cells were seeded in a 6-well flat-bottomed plate, cultured at 37 °C for 24 h and treated with the PKR inhibitor at different concentrations: 500, 1000, 2000, and 3000 nM. DMSO was used as a control. Twenty-four hours after treatment with the PKR inhibitor, proteins were extracted and analyzed with Western blotting. The anti-phosphorylated PKR antibody using in this figure was product number 44–668 G (Life Technologies). Expression of phosphorylated PKR is downregulated in a dose-dependent manner. The bands of phosphorylated PKR are indicated by an arrow, and original gel images of Western blotting are shown in Supplemental Fig. S1 (**A**). Bands indicating total PKR and phosphorylated PKR were quantified, and the phosphorylated PKR:total PKR ratio is shown. The values in the figure represent fold change to those without PKR inhibitor treatment (0 nmol/L), and means ± SEM of three independent experiments are shown. *p < 0.05 compared to the group without the PKR inhibitor by Student’s *t*-test (**B**). To investigate the effects on HCC cell proliferation *in vitro* with the MTS assay, Huh7 cells were seeded in a 96-well flat-bottomed plate with the PKR inhibitor at different concentrations: 500, 1000, 2000, and 3000 nM. Proliferation of Huh7 cells was markedly suppressed by treatment with the PKR inhibitor in a dose-dependent manner. Mean ± SEM of six replicates. *p < 0.05 compared to the group without the PKR inhibitor by Student’s *t*-test (**C**). Photographs of the wells under the microscope at each concentration of the PKR inhibitor (**D**). Scale bar, 200 μm.

Western blotting was performed to examine phosphorylation of PKR after exposure of Huh7 cells, an HCC cell line, to 0, 500, 1000, 2000, and 3000 nM C16. Phosphorylation of PKR was decreased by C16, although total PKR expression remained the same (Fig. 1A). The phosphorylated PKR:total PKR ratio decreased in a dose-dependent manner. Maximum effects of C16 were seen at >2000 nM (Fig. 1B). Phosphorylation of PKR was decreased also with other anti-phosphorylated PKR antibodies (Fig. S2).

Next, the MTT assay was performed to assess division of Huh7 cells treated with 0, 500, 1000, 2000, and 3000 nM C16. Huh7 cell division was decreased by C16 in a dose-dependent manner (Fig. 1C), and the maximum effect was similar to that for phosphorylation of PKR (>2000 nM). No morphological changes in the cells were observed during C16 exposure, suggesting that this inhibitor did not induce substantial toxic effects. The suppressive effects of HCC cell proliferation by PKR inhibitor treatment in a dose-dependent manner were also seen in another HCC cell line, HepG2 (Fig. S3).

### The PKR inhibitor suppressed the growth of HCC cells *in vivo*

To examine the effects of the PKR inhibitor on tumorigenesis *in vivo*, xenograft transplantation experiments were performed using immunodeficient nude mice. Huh7 cells were inoculated subcutaneously into the right flank of nude mice, followed by i.p. injection every day of 0, 30, 100, or 300 μg/kg C16. The volumes of tumors were decreased in the group with C16 injection, and these effects occurred in a partially dose-dependent manner on day 7 after the start of PKR inhibitor treatment (Fig. S4). According to the results, the 300 μg/kg dose of C16 was later used in *in vivo* experiments.

**Figure 2:**
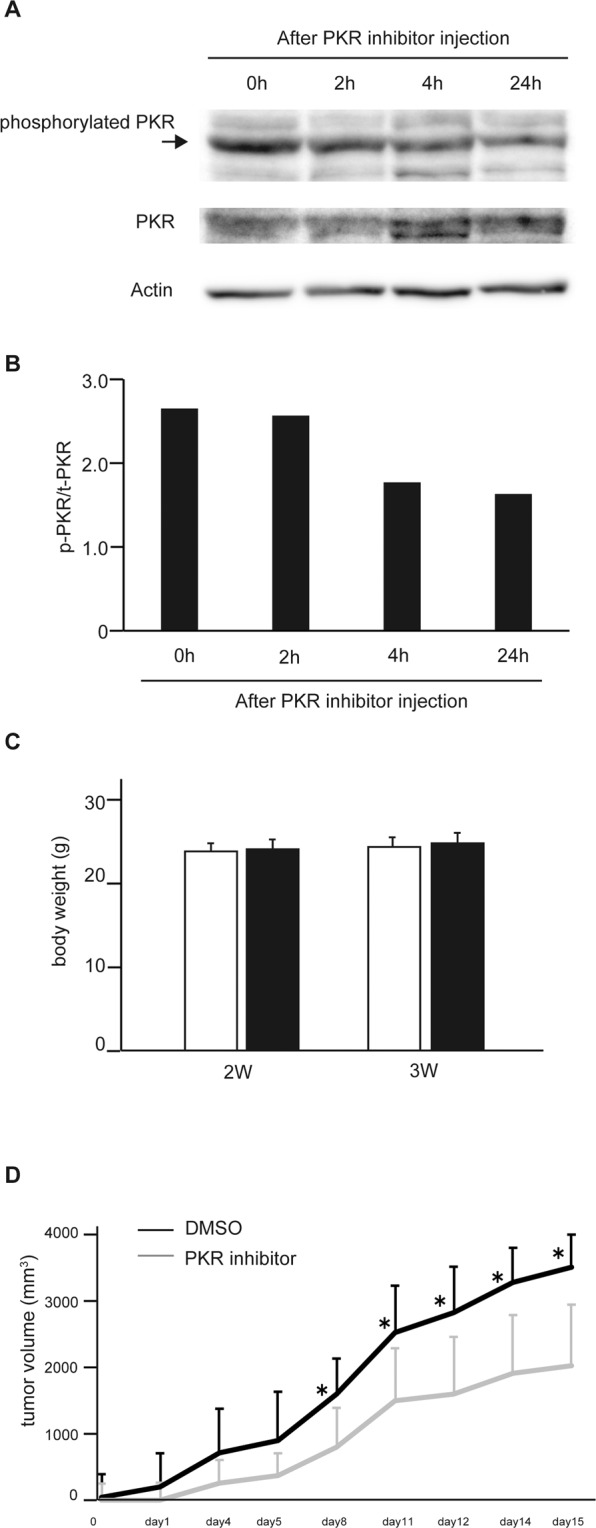
PKR inhibitor suppresses the growth of HCC cells *in vivo* in the xenograft model. Huh7 tumor cells (3 × 10^6^) were inoculated subcutaneously into the flank of female BALB/c-nu/nu mice. The mice were injected i.p. with the PKR inhibitor at 300 μg/kg. Each single xenograft tumor at 0, 2, 4, and 24 h after C16 injection was isolated and each protein sample was extracted. The expression of phosphorylated PKR is suppressed over time after PKR inhibitor injection, as seen with Western blotting. The bands of phosphorylated PKR are indicated by an arrow, and original gel images of Western blotting are shown in Supplemental Fig. S5 (**A**). Bands indicating total PKR and phosphorylated PKR were quantified, and the phosphorylated PKR:total PKR ratio is shown (n = 1) (**B**). Huh7 tumor cells (3 × 10^6^) were inoculated subcutaneously into the flank of female BALB/c-nu/nu mice (n = 6). The groups with and without the PKR inhibitor were injected i.p. every day for 4 weeks with the PKR inhibitor (300 μg/kg) or phosphate-buffered saline as a control. The body weights of the groups with and without PKR inhibitor injection (**C**) and the growth curves of tumor volumes are shown (**D**). The group with PKR inhibitor treatment shows markedly slower tumor growth. Mean ± SEM of six replicates. *p < 0.05 compared to the group without the PKR inhibitor by Student’s *t*-test.

The inhibitory effects of the PKR inhibitor on PKR activation were then confirmed in the *in vivo* xenograft model. Each single xenograft tumor at 0, 2, 4, and 24 h after C16 injection was isolated and each protein sample was extracted. As shown in Fig. 2A, the phosphorylation of PKR was suppressed over time after C16 injection. The phosphorylated PKR:total PKR ratio was suppressed after 4 h, and these effects persisted until at least 24 h (Fig. 2B).

Next, to examine the effects of the PKR inhibitor on tumorigenesis, tumor volume was measured. The body weights of the groups with and without C16 were not significantly different (Fig. 2C). Figure 2D shows the growth curves of tumor volumes after the start of PKR inhibitor injection. The group with C16 injection showed markedly slower tumor growth and smaller tumor size 15 days after the start of C16 injection.

### The PKR inhibitor treatment decreased angiogenesis in HCC tissue in the xenograft model

**Figure 3:**
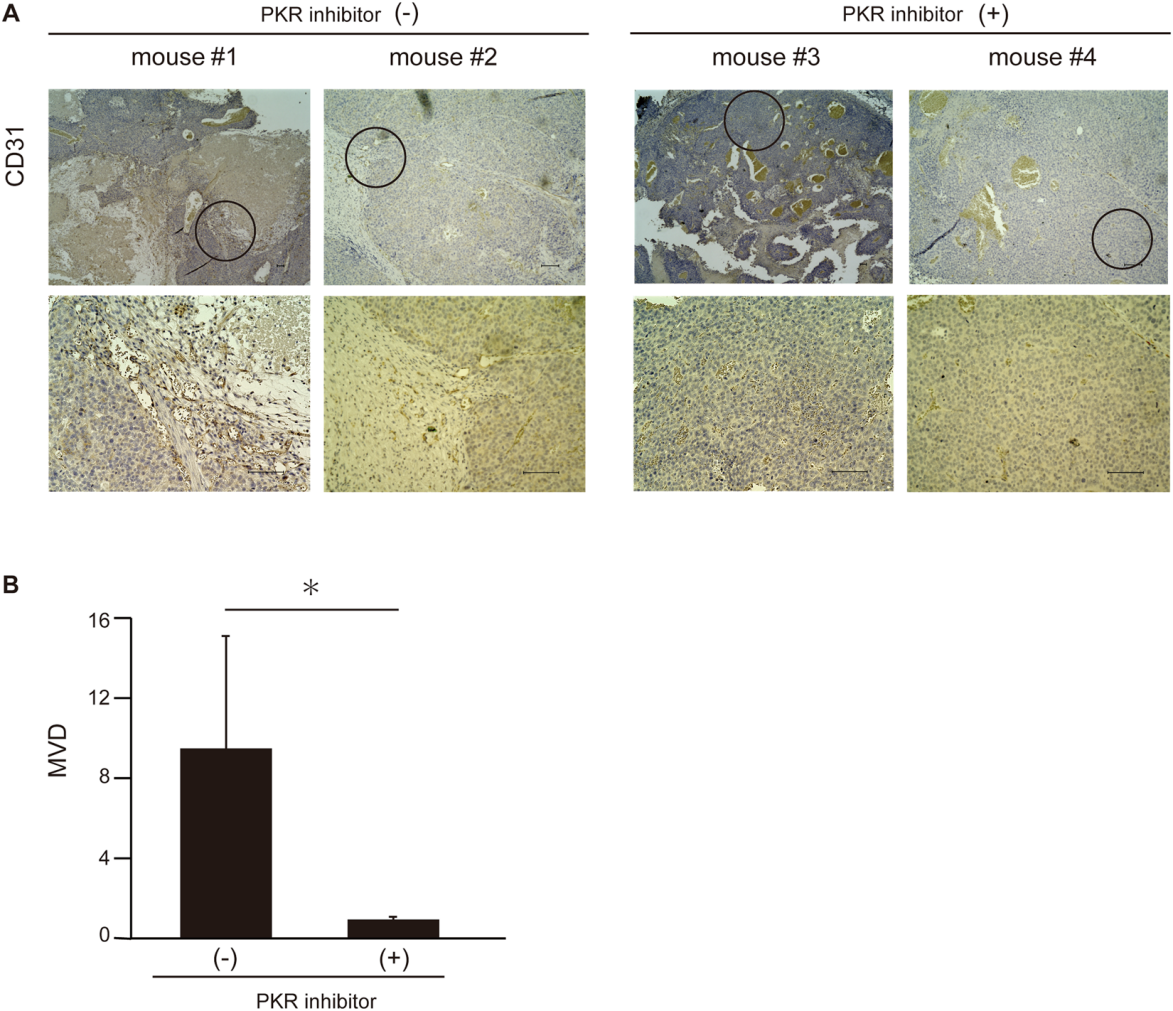
PKR inhibitor treatment decreases the vascularity of HCC tissue *in vivo* in the xenograft model. Immunohistochemical staining for CD31 in isolated xenograft tumors 4 weeks after transplantation (2 weeks after the start of PKR inhibitor treatment). Lower panels show a magnified image of the field indicated by a circle in the upper panels in each mouse. Microvessel density (MVD) measurement was performed as described in the ‘Material and methods’ (**B**). Mean ± SEM of nine areas. *p < 0.01 compared to the group without the PKR inhibitor by the Mann-Whitney U test. Scale bar, 100 μm.

To examine the mechanism underlying the anti-tumor activity of the PKR inhibitor, angiogenesis was investigated in the xenograft HCC model. Four weeks after transplantation (2 weeks after the start of C16 treatment), the mice were sacrificed, and the xenograft tumors were isolated. The tumors of three mice per group with and without C16 injection were examined histopathologically with immunohistochemical staining with CD31 antibody and measurement of microvessel density (MVD). As shown in Fig. 3A,B, MVD in tumor tissues was drastically decreased in the group with C16 treatment compared with the group without C16 treatment. These results indicate that PKR inhibitor treatment caused not only suppression of tumor cell proliferation, but also decreased angiogenesis in HCC tissues.

### The PKR inhibitor downregulated expressions of various growth factors

**Figure 4:**
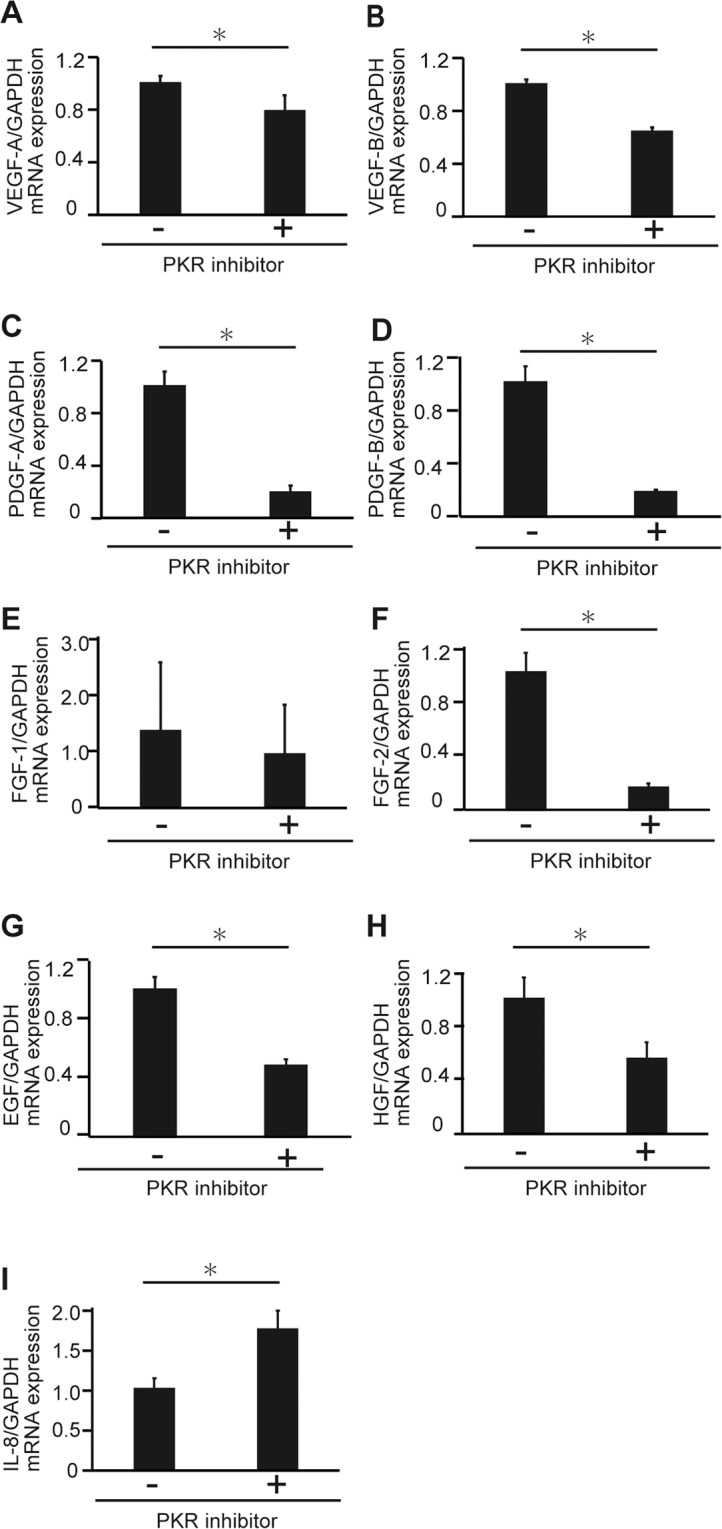
PKR inhibitor treatment downregulates various growth factors in HCC cells. Huh7 cells were treated with 2000 nM PKR inhibitor for 24 h, mRNA expressions of VEGF-A (**A**), VEGF-B (**B**), PDGF-A (**C**), PDGF-B (**D**), FGF-1 (**E**), FGF-2 (**F**), EGF (**G**), HGF (**H**), and IL-8 (**I**) were measured, and the groups with and without PKR inhibitor treatment are compared. Mean ± SEM of six replicates. *p < 0.05 compared to the group without the PKR inhibitor by Student’s *t*-test.

As described before, C16 treatment suppressed the growth of HCC cells, and, moreover, decreased angiogenesis in HCC tissue. To clarify the mechanisms of these phenomena, the effects of C16 on the expression of angiogenesis-related growth factors were investigated. The RT-PCR experiments showed that vascular endothelial growth factor (VEGF)-A, VEGF-B, platelet-derived growth factor (PDGF)-A, PDGF-B, fibroblast growth factor (FGF)-2, epidermal growth factor (EGF), and hepatocyte growth factor (HGF) mRNAs were significantly downregulated by C16 treatment (Fig. 4). In particular, downregulation of PDGF was remarkable (Fig. 4C,D). Interleukin (IL)-8 contributes to angiogenesis and proliferation in various cancers, and we previously reported that PKR positively regulates IL-8 expression in HCV-infected Huh7 cells^16^. However, C16 treatment did not decrease IL-8 expression in HCV non-infected Huh7 cells (Fig. 4I). VEGF-A, PDGF-A, PDGF-B, and EGF expressions were downregulated from the early phase, that is 3 or 6 hours after PKR inhibitor treatment (Fig. S6). These results suggest that downregulation of these growth factors was a direct effect of PKR inhibitor treatment. Similar results were also seen in another HCC cell line, HepG2 (Fig. S7).

## Discussion

The PKR inhibitor used in the present study, oxindole/imidazole compound (C16), was identified as an ATP-binding site-directed small molecule that inhibits the autophosphorylation of PKR^17^. In the field of neurology, loss of PKR increases the late phase of long-lasting synaptic potentiation in hippocampal slices and neurodegeneration in Alzheimer’s disease, and PKR inhibitor treatment rescues these unfavorable states^18,19^. On the other hand, many reports have demonstrated that PKR is overexpressed and activated in several kinds of malignant diseases, such as acute myeloid leukemia, acute lymphoblastic leukemia, colon cancer, breast cancer, and lung cancer^20–23^. Furthermore, as mentioned above, we showed that PKR may have a positive regulatory role in controlling tumor growth and progression in HCC. However, no study has considered the effects of the PKR inhibitor C16 on tumor progression. The present study appears to be the first to show the tumor growth inhibitory effect of C16 treatment in HCC. PKR inhibitor treatment suppressed tumor progression not only by suppression of tumor cell proliferation, but also by decreasing angiogenesis in HCC tissues. Moreover, the mechanism of these effects was found to be suppression of angiogenesis-related growth factors, such as the VEGF, PDGF, FGF, and EGF families.

The activation of proliferative signaling cascades, such as MAPK pathways, by growth factors that bind to receptor proteins activates protein-phosphorylating enzymes and results in the transmission of proliferative signals to the nucleus. While growth factors such as VEGF, EGF, and insulin-like growth factor have been shown to play important roles in liver regeneration after injury, others, such as the PDGF family and FGF, have been implicated in liver fibrosis and HCC growth^24,25^. Solid tumor progression, such as that of HCC, is markedly affected by the development of new blood vessels, or angiogenesis^26,27^. Of the numerous factors that mediate angiogenesis, the VEGF ligand family plays a particularly important role^28,29^. Basic FGF2 is also a potent factor affecting angiogenesis. For example, FGF2 expression has been shown to promote invasion, proliferation, and angiogenesis in numerous types of tumors^30,31^. Furthermore, FGF is overexpressed and activated in HCC, and high levels of FGF2 can potentially be used as a marker of poor clinical outcomes in patients with HCC^31^. EGF/EGFR signaling is known to affect cell differentiation, survival, proliferation, and adhesion^32^, and the overexpression of EGFR has been reported in HCC^33^. HGF is the only known ligand of the receptor tyrosine kinase, c-Met. A recent study showed that the HGF/Met pathway is also involved in tumor invasion, proliferation, and angiogenesis in HCC^34,35^. The induction of PI3K/Akt signaling by PKR, which results in increased choroidal neovascularization and VEGF expression^36^, suggests a connection between PKR signaling and growth factor expression. The findings of the present study demonstrated that the expression of several growth factors was blocked by C16.

In addition, the maintenance of body weight in control and experimental treatments indicated that there were no serious side effects associated with C16 administration. Therefore, C16 could potentially be developed as a novel therapy for HCC.

In a previous study, we demonstrated that the expression and secretion of IL-8 in HCC cells infected with HCV is regulated by PKR^16^. IL-8 is also known to be involved in angiogenesis and the proliferation of some types of cancer^37^. In this study, IL-8 expression was not inhibited by C16. HCV NS5A can modulate IL-8 mRNA and protein expression^38^, and serum levels of IL-8 in patients with chronic hepatitis C have been shown to be significantly higher than those in healthy individuals^39^. It is thus possible that signal transduction in HCC cells may differ depending on whether the cells are infected with HCV.

In another HCC cell line, HepG2, PKR has been shown to increase cell division and migration. For example, in a study involving mice transplanted with HepG2 cells, lentivirus-mediated RNA interference by PKR was shown to have a tumor suppressive effect^40^. In that study, the tumorigenic activities of PKR were attributed to both overexpression of phosphorylated and total PKR in primary tumor tissues relative to the surrounding normal tissues and to STAT3 transcription factor. It is important to note that none of the patients in this study were infected by HBV or HCV, which means that PKR activation also occurs in non-HCV-related liver cancer. These findings are consistent with the present results. However, in the previous report, experiments were performed by downregulating endogenous PKR using shRNA, and thus, therapeutic strategies targeting PKR in HCC could not be fully shown. In addition, the effects of PKR on the microenvironment of tumors were not investigated.

In the present study, C16 treatment had tumor-suppressing effects through inhibition of both tumor cell proliferation and angiogenesis by controlling angiogenesis-related growth factor transcription. C16 is an oxindole/imidazole compound that is an ATP-binding site-directed PKR inhibitor. Further studies examining the role of additional molecules in regulating PKR signaling in liver cancer will provide additional insights into the development of other new small molecule or peptide-based inhibitors as future therapies.

## Materials and Methods

### Ethics statement

All animal experiments were approved by the Ethics Committee for Animal Experiments of Ehime University (#05-RE-2–16). The experimental procedures were conducted in accordance with the approved guidelines.

### Cell culture

The human hepatocellular cell lines Huh7 and HepG2 (Japanese Collection of Research Bioresources, Osaka, Japan) were grown and maintained in Dulbecco’s modified Eagle’s medium (Life Technologies, Carlsbad, CA, USA) supplemented with 10% fetal bovine serum (Life Technologies) and 1% penicillin. Cells were maintained at 37 °C in a humidified atmosphere of 5% CO_2_ and 95% air, and the culture medium was changed three times per week.

### Chemicals

The PKR inhibitor, oxindole/imidazole compound (C16), was purchased from Merck (Darmstadt, Germany) and solubilized in dimethylsulfoxide (DMSO). The final concentrations of the PKR inhibitor in the *in vitro* and *in vivo* experiments are described in the corresponding sections that follow.

### RNA extraction, cDNA synthesis, and real-time RT-PCR

Total RNA was extracted with TRIzol reagent (Life Technologies). Reverse transcription was carried out using the RT-PCR kit (SuperScript^TM^ VILO^TM^ cDNA synthesis kit, Invitrogen, Carlsbad, CA, USA). Real-time PCR was performed using LightCycler technology with SYBR green I dye (Roche Diagnostics, Mannheim, Germany) or Universal Master mix II (Applied Biosystems, Foster City, CA, USA). Commercial VEGF-A (Hs00900055), VEGF-B (Hs00173634), PDGF-A (Hs00234994), PDGF-B (Hs00966522), FGF-1 (Hs01092738), FGF-2 (Hs00266645), EGF (Hs01099990), HGF (Hs00300159), and glyceraldehyde phosphate dehydrogenase (GAPDH) (Hs02758991) (Applied Biosystems) primer sets were used for PCR amplification under the conditions recommended by the manufacturer. For PCR amplification of IL-8, commercial primer sets (Roche Search LC, Heidelberg, Germany) were used under conditions recommended by the manufacturer. GAPDH served as an internal reference gene. The relative mRNA expression levels of host genes divided by the amount of GAPDH mRNA were evaluated statistically.

### Western blotting

RIPA buffer (50 mM HEPES KOH pH 7.9, 150 mM NaCl, 1.5 mM MgCl_2_, and 1.0% v/v NP-40) was added to cells or tumors for protein extraction. Next, 20 µg of protein were separated on 4–12% Bis-Tris Gels (Life Technologies) and transferred to an Immun-Blot PVDF Membrane for Protein Blotting (BIO-RAD, Hercules, CA, USA). Membranes were incubated with the following primary antibodies: anti-beta-actin (Chemicon, Temecula, CA, USA) and anti-PKR (product number: 3210; Cell Signaling, Danvers, MA, USA). Anti-phosphorylated PKR was purchased from Life Technologies (product number: 44–668 G, Figs. 1 and 2), Abcam (Cambridge, England, product number: ab32036; Figs. S2 and S3). Species-specific secondary antibody kits were purchased from General Electric (Charles Coffin, NY, USA). The signal was visualized with an ECL Prime Kit (General Electric). The density of the bands was quantified by normalization to β-actin using Image J Software (National Institutes of Health, Bethesda, MD, USA).

### Cell proliferation assay

*In vitro* cell viability was quantified with the MTS assay (Promega, Fitchburg, WI, USA). Huh7 cells treated with several concentrations (500–3000 nM) of the PKR inhibitor (Merck) were seeded in 96-well plates at 2 ×10^3^ cells per well. At each time point, cells were treated with MTS reagent and incubated for 120 min. Absorbance at 450 nm was recorded. Cells treated with DMSO were used as controls. The wells were photographed under the microscope.

### *In vivo* xenograft transplantation assay

Huh7 cells growing in the logarithmic phase *in vitro* were trypsinized, harvested, washed, and resuspended in Dulbecco’s modified Eagle’s medium with matrix gel (Corning, NY, USA) for tumor implantation. Female 5-week-old BALB/c-nu/nu mice were purchased from CLEA Japan. Then, 3 × 10^6^ Huh7 cells were inoculated subcutaneously into the right flank of nude mice. After confirmation of successful implantation (about 10 mm in diameter), mice were randomly divided into two groups: control (n = 6) and PKR inhibitor injection (n = 6). The group assigned to the PKR inhibitor was injected i.p. every day for 4 weeks with the PKR inhibitor (300 μg/kg), and the control group was injected i.p. with phosphate-buffered saline. For tumor growth analysis, tumor size was measured every day with a sliding caliper, and the tumor volume was defined as (longest diameter) × (shortest diameter)^2^/2. Four weeks after transplantation, the mice were sacrificed using CO_2_, and the xenograft tumors were isolated for use in further experiments. All animal experiments were performed with the approval of the Ehime University Animal Care and Use Committee.

### Immunohistochemical staining and measurement of MVD

Histological diagnoses of formalin-fixed and paraffin-embedded tumor tissues were confirmed on hematoxylin and eosin-stained sections. Sections (5 μm thick) were dewaxed and rehydrated, and then antigen was retrieved by autoclaving for 20 min at 121 °C in EDTA buffer (pH 9.0). The sections were incubated in 1.0% blocking goat serum for 15 min to reduce nonspecific binding. The sections were incubated with primary anti-CD31 polyclonal antibody (Abcam, ab28364, 1:50) at room temperature overnight, followed by the appropriate Histofine Simple Stain Rabbit MAX-PO (Nichirei, Tokyo, Japan) secondary antibody. To quantify MVD, the sections were scanned at low power (×100) to identify the most vascularized areas. Microvessels were counted at ×200 in the three most vascularized areas. A single microvessel was defined as the presence of a lumen composed of cells positive for CD31.

### Statistical analysis

All statistical analyses were performed using SPSS 23 software (SPSS, Chicago, IL, USA). Data are expressed as means ± standard error of the mean (SEM). Significant differences were determined using Student’s *t*-test or the Mann-Whitney U test. P-values < 0.05 were considered significant.

## Supplementary information

Supplementary Information.

## Data Availability

The datasets generated during and/or analyzed during the current study are available from the corresponding author on reasonable request.
