# Supplementary material for: Therapeutic effects of the PKR inhibitor C16 suppressing tumor proliferation and angiogenesis in hepatocellular carcinoma in vitro and in vivo
Source: Sci Rep. 2020 Mar 20;10:5133. doi: 10.1038/s41598-020-61579-x (PMC7083831; doi:10.1038/s41598-020-61579-x)
Supplement: Supplementary file 1 — Supplementary Information. [file 41598_2020_61579_MOESM1_ESM.pdf]

Supplementary Information

Therapeutic effects of the PKR inhibitor C16 suppressing tumor proliferation and angiogenesis in hepatocellular carcinoma *in vitro* and *in vivo*

Takao Watanabe, Hiroko Ninomiya, Takashi Saitou, Sota Takanezawa, Shin Yamamoto, Yusuke Imai, Osamu Yoshida, Ryosuke Kawakami, Masashi Hirooka, Masanori Abe, Takeshi Imamura and Yoichi Hiasa

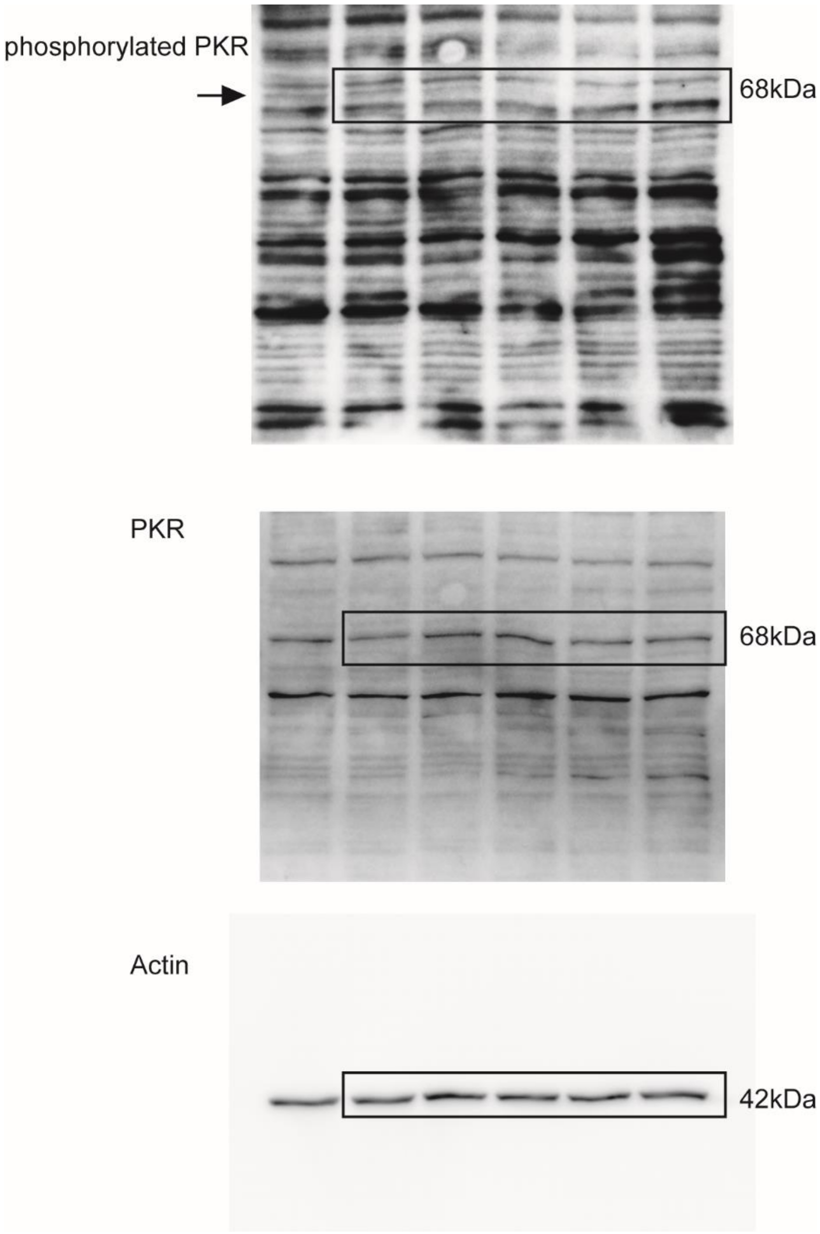

Supplementary Fig. S1. Original blots for Figure 1A.

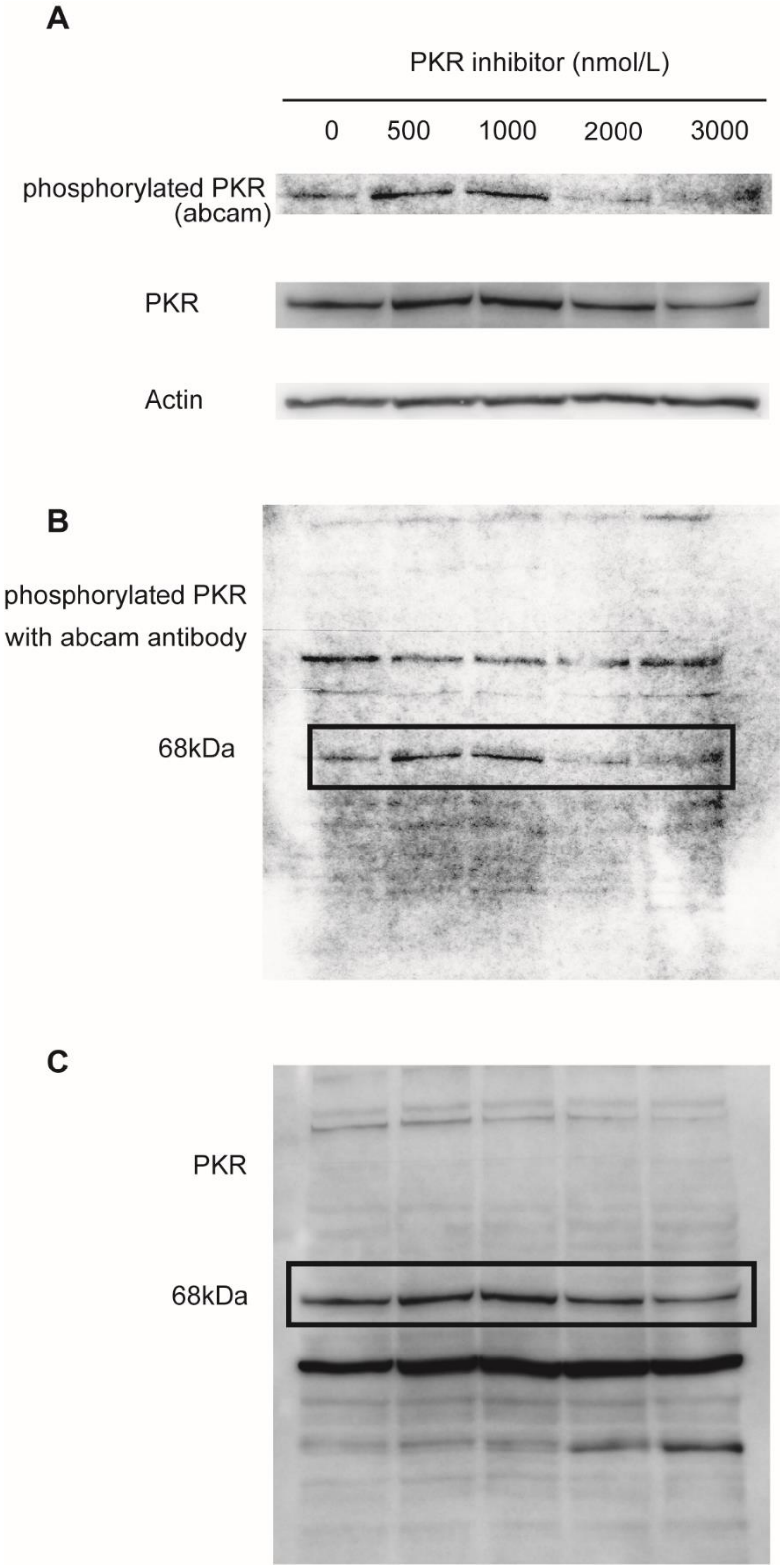
A
PKR inhibitor (nmol/L)
0
500
1000
2000
3000
phosphorylated PKR (abcam)
PKR
Actin
B
phosphorylated PKR with abcam antibody
68kDa
C
PKR
68kDa

Supplementary Fig. S2. Expression of phosphorylated PKR is downregulated by PKR inhibitor treatment with antibodies different from that used in Figure 1.

Huh7 cells were seeded in a 6-well flat-bottomed plate, cultured at 37 °C for 24 h, and treated with the PKR inhibitor at different concentrations: 500, 1000, 2000, and 3000 nM. DMSO was used as a control. Twenty-four hours after treatment with the PKR inhibitor, proteins were extracted and analyzed with Western blotting. The anti-phosphorylated PKR antibody used in this figure was product number ab32036 (Abcam). Expression of phosphorylated PKR is downregulated (A). Original blots for phosphorylated PKR in Supplementary Fig. S2A (B). Original blots for PKR in Supplementary Fig. S2A (C).

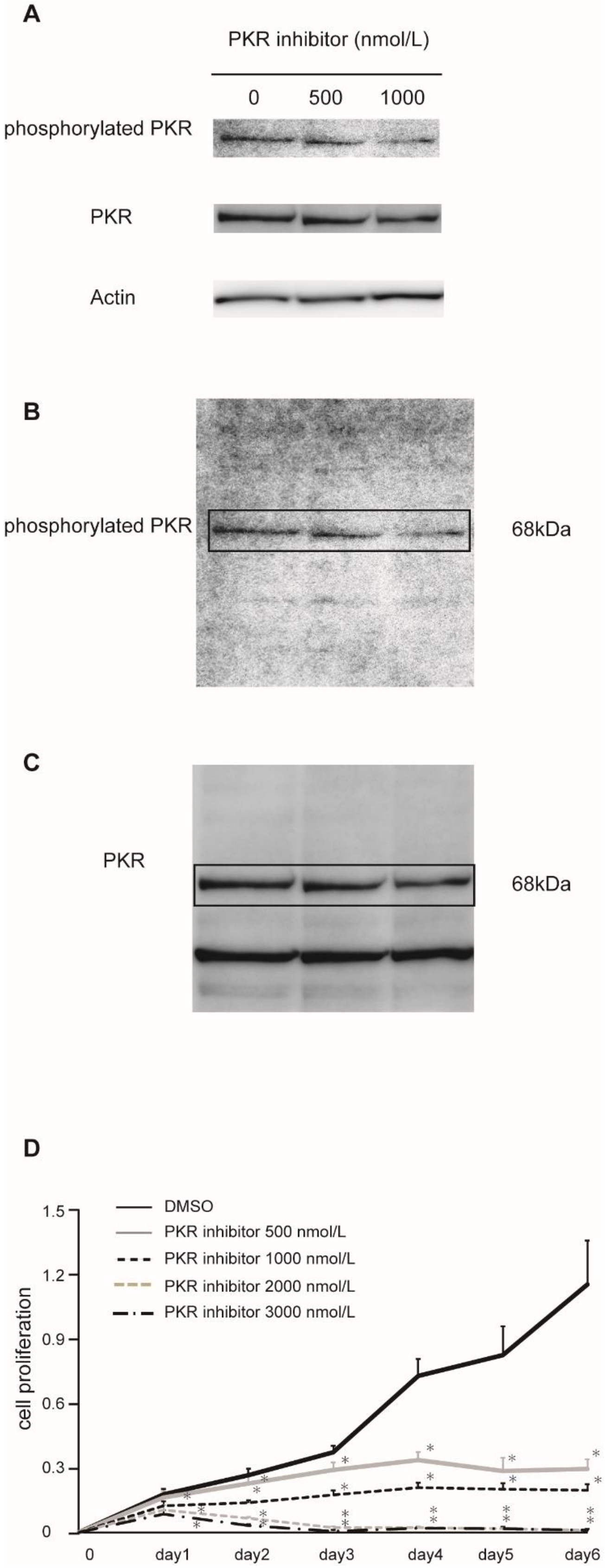
A
PKR inhibitor (nmol/L)
0
500
1000
phosphorylated PKR
PKR
Actin
B
phosphorylated PKR
68kDa
C
PKR
68kDa
D
DMSO
PKR inhibitor 500 nmol/L
PKR inhibitor 1000 nmol/L
PKR inhibitor 2000 nmol/L
PKR inhibitor 3000 nmol/L
cell proliferation
1.5
1.2
0.9
0.6
0.3
0
0
day1
day2
day3
day4
day5
day6

Supplementary Fig. S3. PKR inhibitor treatment suppresses HCC cell proliferation in a dose-dependent manner *in vitro* in HepG2 cells.

HepG2 cells were seeded in a 6-well flat-bottomed plate, cultured at 37 °C for 24 h, and treated with the PKR inhibitor at different concentrations: 500 and 1000 nM. DMSO was used as a control. Twenty-four hours after treatment with the PKR inhibitor, proteins were extracted and analyzed with Western blotting. The anti-phosphorylated PKR antibody used in this figure was product number ab32036 (Abcam). Expression of phosphorylated PKR is downregulated (A). Original blots for phosphorylated PKR in Supplementary Fig. S3A (B). Original blots for PKR in Supplementary Fig. S3A (C). To investigate the effects on HCC cell proliferation *in vitro* with the MTS assay, HepG2 cells were seeded in a 96-well flat-bottomed plate with the PKR inhibitor at different concentrations: 500, 1000, 2000, and 3000 nM. Proliferation of HepG2 cells was markedly suppressed by treatment with the PKR inhibitor in a dose-dependent manner (D). Mean ± SEM of six replicates. *$p < 0.05$ compared to the group without the PKR inhibitor by Student's *t*-test.

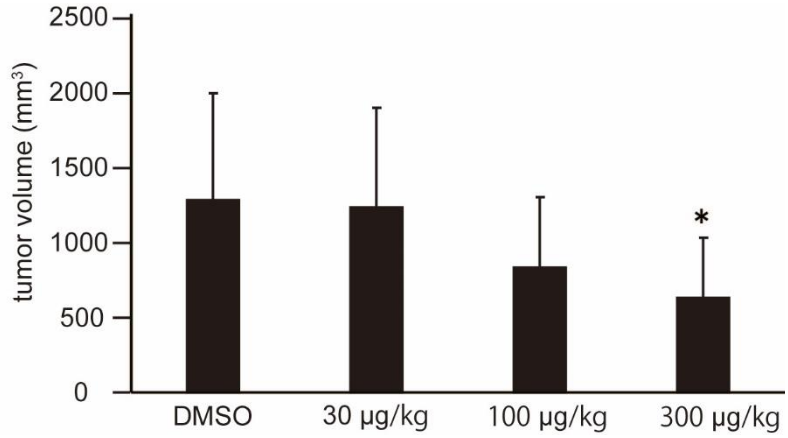

Supplementary Fig. S4. PKR inhibitor suppresses the growth of HCC cells *in vivo* in the xenograft model in a partially dose-dependent manner.

Huh7 tumor cells (3×10^6) were inoculated subcutaneously into the flank of male BALB/c-nu/nu mice (n=4). Mice were injected i.p. with the PKR inhibitor every day at different concentrations, 30, 100, and 300 μg/kg. The tumor volumes on day 7 after the initiation of PKR inhibitor treatment are shown. Mean ± SEM of four replicates. $*p < 0.05$ compared with the control group (DMSO) by Student's *t*-test.

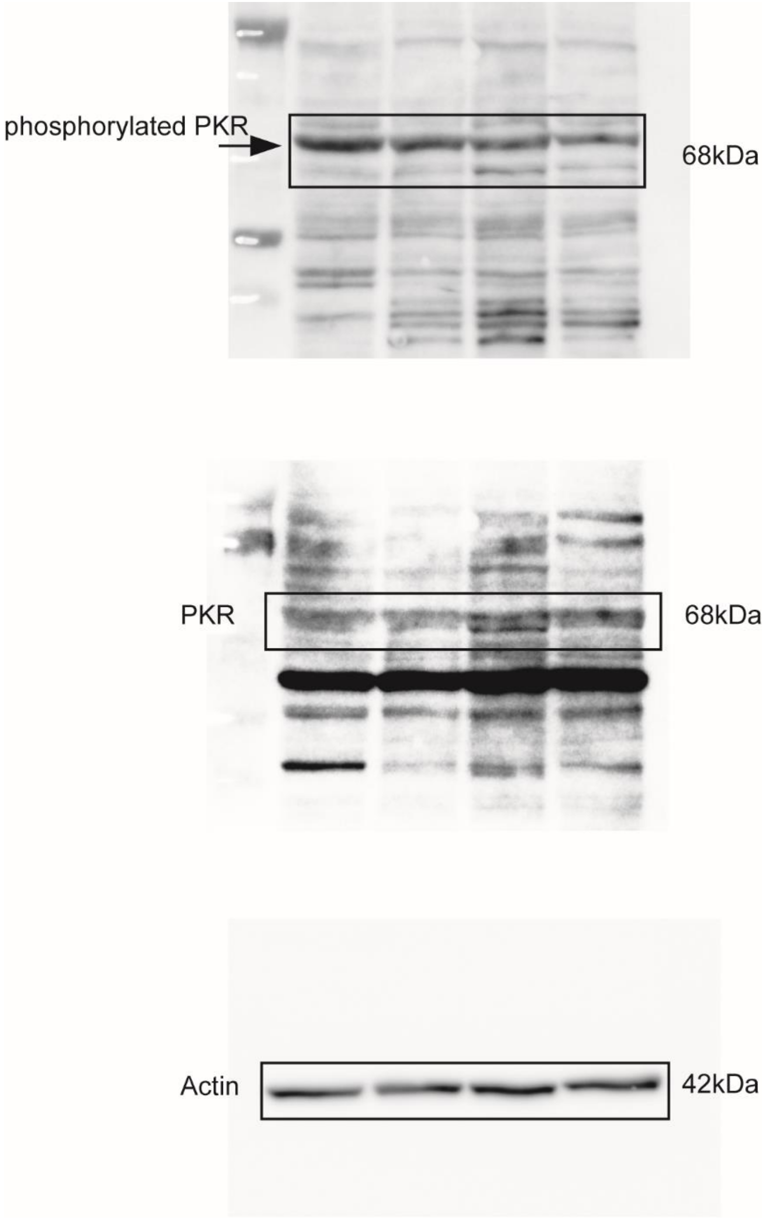

Supplementary Fig. S5. Original blots for Figure 2A.

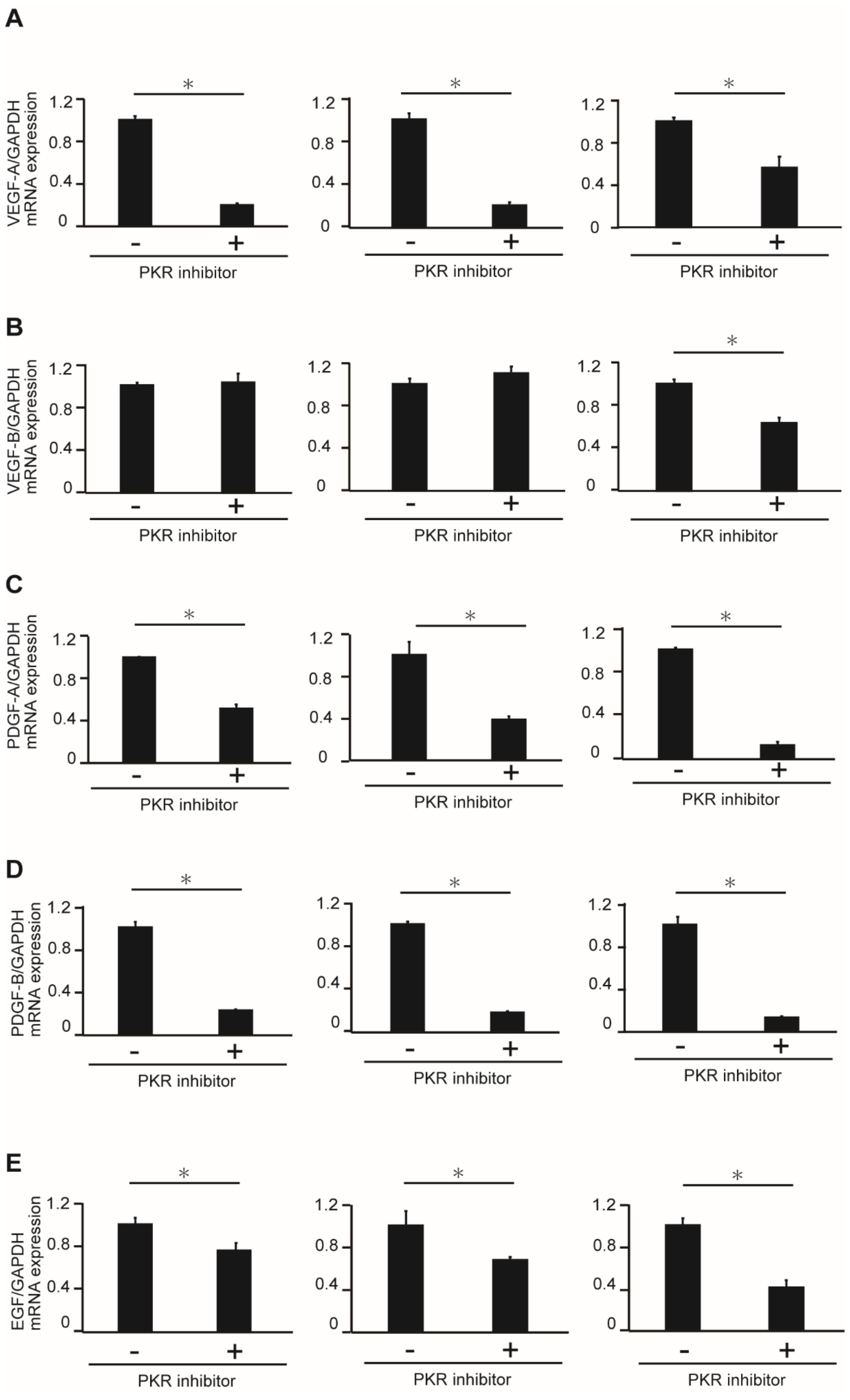
A
B
C
D
E
VEGF-A/GAPDH mRNA expression
VEGF-B/GAPDH mRNA expression
PDGF-A/GAPDH mRNA expression
PDGF-B/GAPDH mRNA expression
EGF/GAPDH mRNA expression
1.2
0.8
0.4
0
*
-
+
PKR inhibitor

Supplementary Fig. S6. PKR inhibitor treatment downregulates various growth factors in Huh7 cells from the early phase after treatment.

Huh7 cells were treated with 2000 nM PKR inhibitor for 3 h, 6 h, and 24 h, mRNA expressions of VEGF-A (A), VEGF-B (B), PDGF-A (C), PDGF-B (D), and EGF (E) were measured, and the groups with and without PKR inhibitor treatment are compared. Left panels show the mRNA expressions of VEGF-A (A), VEGF-B (B), PDGF-A (C), PDGF-B (D), and EGF (E), respectively, 3 hours after PKR inhibitor treatment. The middle panels and right panels show expressions 6 hours and 24 hours after PKR inhibitor treatment, respectively. Mean ± SEM of three replicates. *$p < 0.05$ compared to the group without the PKR inhibitor by Student's *t*-test.

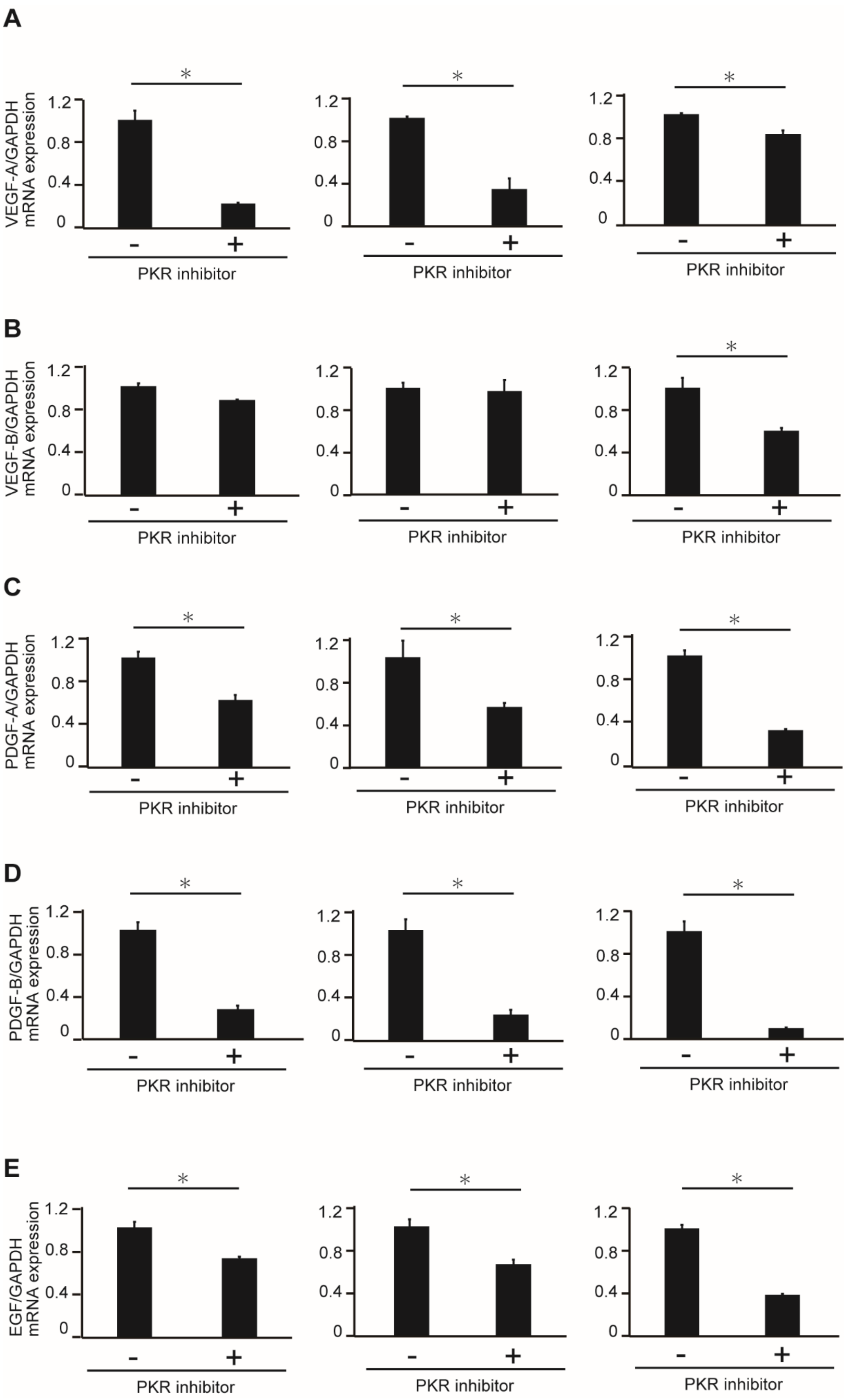

A
VEGF-A/GAPDH mRNA expression
B
VEGF-B/GAPDH mRNA expression
C
PDGF-A/GAPDH mRNA expression
D
PDGF-B/GAPDH mRNA expression
E
EGF/GAPDH mRNA expression
1.2
0.8
0.4
0
*
-
+
PKR inhibitor

Supplementary Fig. S7. PKR inhibitor treatment downregulates various growth factors in HepG2 cells.

HepG2 cells were treated with 2000 nM PKR inhibitor for 3 h, 6 h, and 24 h, mRNA expressions of VEGF-A (A), VEGF-B (B), PDGF-A (C), PDGF-B (D), and EGF (E) were measured, and the groups with and without PKR inhibitor treatment are compared. Left panels show the mRNA expressions of VEGF-A (A), VEGF-B (B), PDGF-A (C), PDGF-B (D), and EGF (E), respectively, 3 hours after PKR inhibitor treatment. The middle panels and right panels show expressions 6 hours and 24 hours after PKR inhibitor treatment, respectively. Mean ± SEM of three replicates. *$p < 0.05$ compared to the group without the PKR inhibitor by Student's *t*-test.
